# Relationship between Behavioural Intention for Using Food Mobile Applications and Obesity and Overweight among Adolescent Girls

**DOI:** 10.3390/ijerph20054432

**Published:** 2023-03-01

**Authors:** Rajaa A. Alyami, Manal F. Alharbi

**Affiliations:** 1 Neonatology Services Improvement Program, General Directorate of Hospitals Affairs, Ministry of Health, Riyadh 11196, Saudi Arabia; 2 Maternity and Child Health Department, College of Nursing, King Saud University, Riyadh 11421, Saudi Arabia

**Keywords:** overweight and obesity, food application, behavioural intention scale, adolescents

## Abstract

Changes in the body mass index (BMI) of children and adolescents have been linked to mobile usage, particularly food applications. This study aimed to investigate the relationship between food application usage and obesity and overweight among adolescent girls. This cross-sectional study was conducted among adolescent girls aged 16–18 years. Data were collected using a self-administered questionnaire from female high schools in five different regional offices across Riyadh City. The questionnaire included questions regarding demographic data (age and academic level), BMI and behavioural intention (BI) scale comprising three constructs: attitude towards behaviour, subjective norms and perceived behavioural control. Of the included 385 adolescent girls, 36.1% were 17 years old, and 71.4% had normal BMI. The overall mean BI scale score was 65.4 (SD 9.95). No significant differences were observed between overweight or obesity in relation to the overall BI score and its constructs. A high BI score was more associated with participants studying in the east educational office than those who were enrolled in the central educational office. Behavioural intention to use food applications greatly influenced the adolescent age group. Further investigations are necessary to determine the influence of food application services among individuals with high BMI.

## 1. Introduction

Childhood obesity is one of the most challenging global health issues of the 21st century [1]. It has been established that body weight during childhood directly impacts an individual’s lifelong health [2]. According to the World Obesity Atlas 2022, published by the World Obesity Federation, one billion people will be obese by 2030. This includes one in five women and one in seven men [3]. Globally in 2016, over 340 million children and adolescents aged up to 19 years were overweight or obese [4]. Adolescents, a group comprising individuals who are 10–19 years of age, are of interest as they undergo various physical, sexual, psychological and social developmental changes [5]. Contrary to popular belief that individuals in this age group are often healthy, adolescents face several health issues and are at high risk of being overweight and obese until their adulthood and developing noncommunicable diseases (NCDs) like diabetes and cardiovascular diseases at a young age [4,5]. The body mass index (BMI) is used to diagnose childhood overweight and obesity. Overweight is defined as having a BMI at or above the 85th percentile and below the 95th percentile for children and adolescents of the same age and sex, whereas obesity is defined as having a BMI at or above the 95th percentile for children and teens of the same age and sex [6]. Increasing levels of overweight and obesity pose some risks to the health and well-being of children and adolescents [7]. Technology has led to an increase in overweight and obesity [8]. As a type of technology used to deliver food online, food apps may pose serious health risks. Therefore, prevention of weight gain among children and adolescents requires taking food apps into consideration as risk factors. Further research is required to understand these implications.

### 1.1. Literature Review

Obesity is a major problem in the USA, with its prevalence being higher in adolescents than in children of other age groups. According to the Center for Disease Control and Prevention (CDC), the prevalence of obesity is 13.9% among 2–5-year-olds, 18.4% among 6–11-year-olds and 20.6% among 12–19-year-olds [9]. It is known that being overweight and obese status are caused by a variety of factors, including eating patterns, lack of sleep or physical activity, certain medications, and genetic factors. [10]. A systematic review revealed the association between friendship networks and obesity-related behaviours among adolescents [11]. A study in Iraq revealed that overweight and obesity were higher among females than among male adolescents [12]. In Saudi Arabia, the overall prevalence figures of overweight and obesity were 13.4% and 18.2%, respectively, and when compared with the WHO-based national prevalence rate of obesity reported in 2004 (≈9.3%), the obesity rate has doubled in 10 years [13]. Also, the estimated prevalence rates of overweight and obesity among school-aged children were 19.6% and 7.9%, respectively, and high rates were reported for adolescents (26.6% and 10.6% for overweight and obesity, respectively) [13]. Many studies have identified overweight and obesity as health issues in Saudi Arabia [14,15,16,17] and observed a direct relationship between obesity and several factors, such as skipping breakfast, excessive calories, consumption patterns, and parental socioeconomic factors [16,17,18,19].

There are many factors that may influence the development of obesity, such as lack of physical activity and digital devices [20]. Digital device use has been associated with many evidence-based benefits, including early learning, exposure to many ideas and knowledge, enhanced social interactions and support and increased access to health promotion information and messages [21]. However, the use of such digital devices can compromise sleep, attention, and learning; increase the incidence of obesity and depression; and expose people to inaccurate, insensitive, or unsafe information [21]. In contrast, a recent study suggests that digital devices can empower parents who are concerned about their children’s overweight and obesity [22]. Studies have found an increased incidence of high BMI in children and adolescents who use digital devices [23,24,25,26,27], including ordering food online. Also, the use of online food ordering systems is increasing [21]. Currently, more than 1.2 billion people use online food delivery systems (OFDs) worldwide. By 2027, the total number of people using platform-to-consumer delivery systems will reach 1517.4 million [28]. Within the past four years, food orders placed through direct restaurant apps or third-party services have increased by 23% in the USA [29]. The convenience of food applications can lead to adverse health outcomes. In addition to being easy to use, these apps provide access to menus and offers available at local restaurants. This demonstrates how technology has a significant impact on lifestyle and wellness. For instance, in the U.S., there is a lack of research to support how digital food ordering affects health and wellness from an individual or public health perspective [29], and also, studies that investigate if the users of food applications are overweight or obese or among overweight or obese adolescents. Additionally, an early report reported that the obesity prevalence is expected to rise from 12% in 1992 to 41% by 2022 for men and from 21% to 78% for women, according to World Health Organization projections for 1992 to 2022 in Saudi Arabia [30]. This highlights the critical gaps in the literature regarding the investigation of the relationship between food apps and obesity among adolescence. More research is needed to explain the underlying mechanisms and provide effective prevention strategies [20].

Considering food apps as risk factors in both age groups helps in applying preventive measures and decreasing weight gain among children and adolescents. In order to fully understand the potential health implications of online food delivery, further research is needed. The current research on the impact of digital use on lifelong health is lacking. As a result, and because of this percentage among female adolescents, it is imperative to predict and recognise target-oriented behaviour, take preventive measures, and raise awareness among young women about their health. Therefore, this study aimed to assess the intended behaviour of using food apps by applying the theory of planned behaviour (TPB).

### 1.2. The Conceptual Framework

The theory of planned behaviour is one of the social psychology theories that are widely used in health promotion activities. TPB can offer a reasonable explanation of the decision-making processes underlying both the intention for and engagement in self-care overweight/obesity-reducing behaviours [31]. It believes that people are rational and their decisions are based on the knowledge available to them. TPB explains and understands environmental and individual factors that influence behaviour. The important determinant of a person’s behaviour is their intention [31]. Three interrelated concepts are described in TBP, and these concepts can serve as factors that define the level of behavioural intention (BI) [32]. First, attitude towards the behaviour (ATB) refers to the individual’s positive or negative evaluation of performing the behaviour [32]. Attitude is perceived as a combination of feelings, beliefs, intentions and perceptions. Second, subjective norm (SN) is viewed as the social pressure upon a person to behave in a certain way [32]. Lastly, perceived behavioural control (PBC) is related to the perceived influence of factors on the behaviour; therefore, it may enhance or hamper certain behaviours [32]. Thus, the intention should be placed at the core of an individual’s behaviour to act upon the three concepts [32]. Accordingly, positive ATB and SN lead to high perceived control PBC and a strong individual intention to perform a positive behaviour [32].

**Figure 1:**
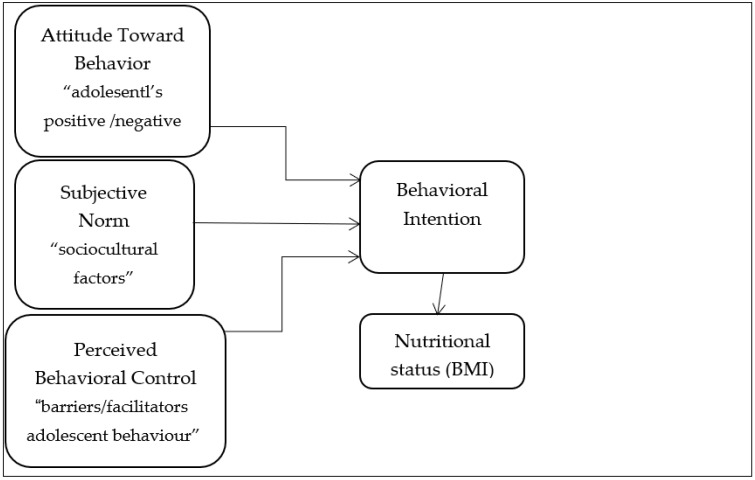
Conceptual framework of the current study. The proposed model illustrates the relationships between each of the variables in the model of the theory of planned behaviour.

For the current study, using food apps, the attitude towards an action or behaviour predicts that participants might believe that using an app is more convenient. SN focus on the surroundings of the individual, such as family, friends, beliefs, habits or social media advertising that probably influence their decision. The PBC means that adolescents respond to using apps as an easy way to fulfil their diet needs. Because the theory helps predict a positive or negative attitude towards an action, if the three concepts are positive or two of them are, the intention is increased. Therefore, adolescents will be more likely to have the behaviour of using food apps. See Figure 1.

Behavioural intention for using food apps may influence BMI. Attitude towards the behaviour, sociocultural factors and barriers associated with facilitators of adolescent behaviour influence behavioural intention to food app user behaviour. Therefore, recognising the food app intention behaviour as a factor that leads to overweight and obesity will be useful in developing prevention measures. Thus, this study aimed to investigate the influence of food app intention behaviour on adolescent girls in Saudi Arabia using the theory of planned behaviour (TPB). In addition, the current study hypothesised that the attitude towards the behaviour, subjective norm, and perceived behavioural control predict behavioural intention.

## 2. Subjects and Methods

### 2.1. Design and Participants

This study adopted a quantitative descriptive design. This design was selected to test the hypothesis of the study. It is a deductive approach where concepts of obesity, overweight and food apps are downed to variables, and their relationship is tested. When an evidence-based conclusion is drawn, generalisations can be extended to a larger population. Given the cross-sectional design of our study, the variables and relationships among them were determined [33]. Data were collected from female high schools in five different educational regions across Riyadh City. This study included female students aged 16–18 years. The required sample size was determined as 383, which was determined on the basis of the estimated population size of 86,704 obtained from the Ministry of Education database that was last updated in June 2016 [34]. Calculations were made using Epinfo version 7.2.3, with a confidence level of 95%, a margin of error of 5%, an expected frequency of 50% and a design effect of 1.0 in five clusters [35]. This sample size was increased by approximately 15% (415) to compensate for any absenteeism, dropouts or incomplete questionnaire. Thus, the returned sample was 390 after excluding five incomplete questionnaires.

### 2.2. Data Collection

The study was conducted from January to March 2021. Quantitative data were collected by distributing a self-report questionnaire and performing physiologic measurements of the student’s weight and height to evaluate their BMI. In order to ensure the most desirable representative sample, probability sampling was conducted, which entails both clustering and stratified sampling techniques. The sample was divided into five clusters that are distributed based on five regions of Riyadh City. Each regional cluster involved one randomly selected high school. Moreover, stratified sampling was applied to the selection of classrooms. Since female high schools were accessible, all female students were grouped by level (first, second, and third). From each level, one class was selected randomly. All students in the selected classes were recruited [33]. Approximately 79 students in each regional sample cluster were enrolled. Data collection followed specific ethical protocols that involved an explanation of the purpose of the study to the participants and the distribution of questionnaires by researchers, ensuring voluntary participation and an agreement of participation secured. The questionnaires were handled confidentially, and all collected data were manually verified. BMI was measured using a formula after taking the participants’ weight and height. The CDC recommends BMI categorisation for children and teens between ages 2 and 20 years. Therefore, the BMI-for-age percentile growth charts were used. The CDC BMI categorisation for children and teens between the ages of 2 and 20 is as follows: underweight, <5%; healthy weight, 5%–85%; at risk of overweight, 85%–95% and overweight, >95% [36].

### 2.3. Measurements

A 24-item questionnaire that consists of close-ended questions was developed by the researchers. The self-administered questionnaire consisted of four parts. Each part assessed a certain variable. Part one assessed the sociodemographic characteristics of the adolescent. Part two assessed ATB with ten items (i.e., adolescent attitudes towards the use of food apps). Part three assessed SN with four items (i.e., adolescent sociocultural factors). Finally, part four assessed PBC with six items (i.e., barriers and facilitators of adolescent behaviours). In order to measure these variables, a 5-point Likert scale was used for all parts, with positively worded statements and various response options. These options, as a form of frequency that ranges from always to never, an agreement that ranges from strongly disagree to strongly agree and a level that ranges from very high to very low. Positive statements are scored 1–5. Each score item was reported individually [33].

### 2.4. Validity and Reliability

It is necessary to measure the validity and reliability of the research established scale. Content validity evaluates whether questions cover all aspects of the study and whether irrelevant questions will be removed [33]. An empirical method of testing content validity involves techniques to calculate the content validity index (CVI). The CVI was determined by experts (*n* = 7) from the field who evaluated each item of the questionnaire. The items were evaluated for their clarity, relatedness, representativeness and appropriateness, and their instructions were suited for the target group. Each item uses a four-point scale (from 1 as not relevant to 4 as very relevant) to determine whether the item is to be approved or rejected [37]. The rating scales were described on the item level (I-CVI) and scale level (S-CVI). I-CVI was measured by I-CVI = (agreed item)/(number of experts). S-CVI was determined by which the scoring average of the I-CVI for all items on the scale (S-CVI/Ave) and the item’s proportion on the scale that scored a scale of 3 or 4 by all experts (S-CVI/UA) [38,39]. In order to test the reliability of the established scale, the scale requires examination of the stability and internal consistency as the best and oldest technique used for consistency. Coefficient alpha interpretation constitutes the normal range of values that is between 0.00% and 1.00%, and higher values indicate a greater internal consistency [33]. The established scale demonstrated properties of reliability for the adolescent age group. The internal consistency calculated using Cronbach’s alpha was found to be good (α = 0.84) for 20 items of the entire scale (BI), subscales ATB (α = 0.80) and PBC (α = 0.71). Other categories showed acceptable levels for the subscale SN (α = 0.66). Additionally, the average inter-item correlation calculated for determining the appropriate internal consistency reliability was good (0.42) and showed a positive and had a good item level, ranging between 0.20 and 0.57. The ideal range for item level is considered to be 0.15–0.50, and values over 0.2 are considered acceptable [40,41].

### 2.5. Statistical Analysis

The IBM SPSS for Windows, version 26.0 (IBM Corp., Armonk, NY, USA), was used to analyse all data. Descriptive statistics were presented using numbers, percentages and mean ± standard deviation. Sociodemographic characteristics and BI were compared using the Kruskal–Wallis test, whereas differences in the score of BI and its constructs according to the BMI level were analysed using the Mann–Whitney U-test. Furthermore, Spearman’s correlation coefficient was used to determine the correlation between the BI scale and its constructs. The normality test was conducted using the Shapiro–Wilk test and Kolmogorov–Smirnov test. A *p* < 0.05 was taken as significant.

## 3. Results

A total of 385 young girls completed the survey, and five incomplete questionnaires were excluded. As shown in Table 1, the most common age was 17 years (36.1%). Girls enrolled from the north educational office constituted 20.5%, of which 36.6% of them are currently in the second year. Additionally, participants with normal BMI were predominant (71.4%). Table 2 shows the mean score of the BI constructs, which are composed of the ATB, SN and PBC. Regarding the ATB, the mean score was highest in the statement ‘I like to use the food app because it is easy and convenient’ (mean score, 3.76), followed by ‘Food apps give various food choices than home meals’ (mean score, 3.67) and ‘Meals that are ordered through food apps are more attractive than home meals’ (mean score, 3.56), whereas it was lowest in the statement ‘I used to order through food apps almost daily’ (mean score, 2.45). The overall mean score was 31.6 (SD 5.82). For the SN, the mean rating was the highest for the statement ‘Food apps advertisement is everywhere’ (mean score, 4.34), whereas it was lowest in the statement ‘Food apps were recommended by my friends’ (mean score, 3.12). The overall mean score for SN was 14.8 (SD 2.49). Finally, for the PBC, the mean score was highest for the statement ‘It is easy to order food through food apps because I have a device’ (mean score, 3.97), followed by ‘It is easy to order food through food apps because I have internet access all the time’, whereas it was the lowest in the statement ‘It is easy to order food through food apps because my mother is busy and cannot cook home meals’ (mean score, 2.30). The overall mean score of the PBC was 18.9 (SD 4.25) and the mean total BI score was 65.4 (SD 9.95). In Table 3, a positive and highly significant correlation was found between BI scores among its constructs, including ATB (r = 0.870), SN (r = 0.682) and PBC (r = 0.750). In addition, we noted a positive and highly significant correlation between attitude towards behaviour according to SN (r = 0.469) and PBC (r = 0.394). Finally, a positive and highly significant correlation was observed between SN and PBC (r = 0.369). In Table 4, no significant correlation was found between the BMI level and the total BI score, including its constructs such as ATB, SN and PBC (*p* > 0.05). In Table 5, a higher BI score was more associated with the east educational office, but it was the lowest in the central educational office (H = 28.813; *p* < 0.001). No significant differences were found in BI according to the age group (*p* = 0.099) and academic year level (*p* = 0.274). In Table 6, the post-hoc analysis indicates that the mean differences in BI scores were significant between the south educational office and the east educational office (*p* = 0.015). Moreover, we found significant differences between the central educational office and the north educational office (*p* = 0.002), west educational office (*p* = 0.007) and east educational office (*p* < 0.001).

## 4. Discussion

This study investigated the relationship between food apps among adolescent girls who are overweight and obese. To our knowledge, this is the first study in Saudi Arabia-Riyadh city that tested the influence of ordering meals through digital apps on the weight levels of female high school adolescents. We employed the TPB as a tool for measuring behaviour intentions in using digital food apps. The results of this study revealed that the overall BI score has a mean of 65.4 (SD 9.95). Regarding its constructs, the ATB mean score was 31.6, the SN mean score was 14.8, and the PBC mean score was 18.9. The overall scores of the BI and its constructs were above the average of the mean points, suggesting the high behavioural intention to use food apps among the participants. The continuous innovation of the digital world is reflected even in food consumption, and the increased behaviour of utilising food apps was evidently seen in our results. This study adds to the existing discussion on consumer behaviour in the context of digital food delivery in Saudi Arabia and uncovers the elements that could be used to predict people’s motivation to buy food through food delivery apps.

A positive and highly significant correlation was found between the overall BI score and its constructs, suggesting that the increase in the score of each ATB, SN and PBC construct correlated with the increase in the overall BI score. For instance, increasing adolescents’ attitudes, SN or PBC to ordering food through food apps correlated with an increase in overall behavioural intention. Consistent with our findings, Choyhirun et al. (2008) found that attitudes, SN and PBC explained up to 41.8% of the variance in intentions [42]. The intentions were influenced most by PBC and then by attitudes and SN. The positive trend of behavioural intentions in using food applications among our youth may lead to overconsumption, which may result in unhealthy food consumption. This scenario may be in accordance with that of Lwin et al. (2017) as well as Andrews, Silk and Eneli (2010) [43,44]. According to their reports, the enhanced attitude of children towards eating healthy food is directly influenced by the guidance of parents, decreasing the intention to eat unhealthy food. However, parental mediation of TV advertising negatively affected healthy food attitudes to a greater extent [43,44]. Hence, parental guidance to children is imperative when ordering food through apps to avoid overconsumption of food and an unhealthy lifestyle.

Our results suggest that although participants who were overweight/obese had a slightly higher attitude in using food apps, adolescents with normal/underweight BMI had slightly higher SN, PBC and behavioural intentions; however, this did not yield significance (*p* > 0.05). In a study conducted in Thailand among 112 overweight (*n* = 52) and obese (*n* = 56) young adults, the overall mean TBP score increased significantly from baseline in the health dieting behaviour and SN following group counselling, concluding that group counselling was not inferior to individual counselling and that group counselling is a better option for healthy dieting management [45]. In Greece, between the normal-weight group and overweight/obese group, correlations between variables of TPB and behaviours (healthy eating and exercise) were higher in the normal-weight group than in the overweight/obese group, whereas attitude was a significant predictor for those with higher values in the normal-weight group [46]. On the contrary, several studies have reported a decrease in BMI after educational interventions. For example, Jejhooni et al. (2022) reported that before the educational intervention, no significant difference was found in the behavioural intention between the experimental group and control group; after six months of the training intervention, a significant increase was found in each of the TPB constructs, weight and BMI among the intervention group whereas the control group did not differ significantly after the educational intervention [47]. This has been concluded by Sanaeinasab et al. (2020), Mazloomy-Mahmoodabad et al. (2017) and Soorgi, Miri and Sharifzadeh (2015), revealing significant changes after educational intervention in behavioural intentions and BMI levels specifically towards the experimental group [48,49,50].

No significant differences in BI were found in relation to age and academic year level (*p* > 0.05). These findings are similar to those of Jeihooni et al. (2022) [47]. No significant difference was found in their study in TBP constructs at baseline between the experimental and control groups in terms of age and education. The study by Alfadda and Masood (2019) correlated overweight and obesity with high levels of parental socioeconomic status and urbanisation in Saudi society [18]. Although caution may be warranted, further investigations should be conducted to determine the effect of behavioural intentions on the sociodemographic data of the overweight and obese populations. One unanticipated finding was the difference between BI, and educational region office location was significant (*p* < 0.001). The east educational office was associated with a higher BI score, whereas the central educational office was associated with a lower BI score. A possible explanation for these results may be the lack of adequate data on the financial and employment status of the participants and their parents or living in a location that has a variety of restaurants, which causes a high intrinsic motivation to order using food application. A limitation of this study is that descriptive studies are not helpful in understanding the causes of the phenomena, as the survey method limits the ability to identify causes. Additionally, there is a lack of information regarding the use of food apps by boys in high schools. Despite these limitations, this study can help in testing a newly established scale. Although the scale revealed good reliability and validity characteristics, further explorative and validation studies should be considered to test the newly established scale. Further research is recommended to establish the influence of food app services among individuals with increased BMI levels. It would be a fruitful endeavour for the current findings to be repeated in different contexts for a better understanding of the factors that influence the intended behaviour of food app use in a larger age group. Moreover, this study can serve as a reference guide and baseline for research investigating such topics in the future.

## 5. Conclusions

This study set out to investigate the influence of food app intention behaviour on adolescent girls in Saudi Arabia using the theory of planned behaviour (TPB). This study has found that, generally, the behavioural intention to use food apps greatly influences our sample population. There was a high behavioural intention among the participants to use food apps, as indicated by the overall scores of the BI and its constructs. This was above the average of the mean points. As the digital world develops, so does food consumption, and our results demonstrate that more people are taking advantage of food apps to consume food. Through food delivery apps, we explore the elements that could be used to predict people’s motivations to buy food in the context of digital food delivery in Saudi Arabia. The results indicate that although adolescents with overweight/obese BMI had a slightly higher attitude towards using food apps, adolescents with normal/underweight BMI had slightly higher SN, PBC, and behavioural intentions. Further, evidence suggests that ATB, SN, PBC and overall BI were not directly related to the increased weight of the young population. However, the increase in the overall behavioural intention to use food apps could be associated with participants enrolled in the east educational office but less among those enrolled in the central educational office.

## Figures and Tables

**Table 1:** Sociodemographic characteristics of the participants (*n* = 385).

Study Variables	N (%)
Age group	
16 years	124 (32.2%)
17 years	139 (36.1%)
18 years	122 (31.7%)
Educational region office	
South educational office	78 (20.3%)
North educational office	79 (20.5%)
West educational office	78 (20.3%)
Central educational office	76 (19.7%)
East educational office	74 (19.2%)
Academic year level	
First year	124 (32.2%)
Second year	141 (36.6%)
Third year	120 (31.2%)
BMI level	
Underweight (<5th percentile)	36 (09.4%)
Normal (5th–<85th percentile)	275 (71.4%)
Overweight (85th–<95th percentile)	38 (09.9%)
Obese (≥95th percentile)	36 (09.4%)

**Table 2:** Mean score of the behavioural attention constructs (*n* = 385).

Behavioural Attention Statements	Mean ± SD
1. I like to use the food app because it is easy and convenient	3.76 ± 0.90
2. Food apps can rapidly ease my hunger	3.31 ± 1.03
3. Food apps help me save time	3.67 ± 0.98
4. Food apps give various food choices than home meals	3.49 ± 1.07
5. I prefer to order meals through food apps than eating home meals	3.01 ± 1.01
6. Food apps meals’ quality and taste are the same as home meals	2.48 ± 0.85
7. Meals that are ordered through food apps are more attractive than home meals	3.56 ± 0.99
8. I used to order through food apps almost daily	2.45 ± 0.85
9. I used food apps many times per week	2.79 ± 1.02
10. Meals that are ordered via food apps can fulfil my dietary needs	3.12 ± 0.98
Attitude towards behaviour	31.6 ± 5.82
11. Food apps were recommended by my friends	3.12 ± 0.94
12. Food apps advertisement is everywhere	4.34 ± 0.72
13. My friend uses food apps	3.65 ± 0.93
14. My family members use food apps	3.65 ± 0.93
Subjective norms	14.8 ± 2.49
15. My family income allows me to order meals through food apps	3.49 ± 1.04
16. My allowance enables me to order meals through food apps	3.17 ± 1.15
17. It is easy to order food through food apps because I have internet access all the time	3.72 ± 1.09
18. It is easy to order food through food apps because I have a device	3.97 ± 0.97
19. My family members encourage me to order meals through food apps	2.34 ± 1.07
20. It is easy to order food through food apps because my mother is busy and cannot cook home meals	2.30 ± 1.25
Perceived behavioural control barriers	18.9 ± 4.25
Total behavioural attention score	65.4 ± 9.95

Note: response ranges from 1 (strongly disagree) to 5 (strongly agree).

**Table 3:** Correlation between attitude towards behaviour, subjective norms, perceived behavioural control and the total behavioural attention scales (*n* = 385).

Behavioural Attention Parameters	BA	ATB	SN	PBC
Behavioural attention (BA)	1			
Attitude towards behaviour (ATB)	0.870 **	1		
Subjective norms (SN)	0.682 **	0.469 **	1	
Perceived behavioural control (PBC)	0.750 **	0.394 **	0.369 **	1

** Correlation is significant at the 0.01 level (two-tailed) using the Spearman Correlation test.

**Table 4:** Differences in the scores of the behavioural attention and its constructs according to BMI level (*n* = 385).

Behavioural Attention	Normal or Underweight Mean ± SD	Overweight or Obese Mean ± SD	*p*-Value *
Attitude towards behaviour	31.6 ± 5.69	31.8 ± 6.36	0.882
Subjective norms	14.8 ± 2.38	14.6 ± 2.94	0.450
Perceived behavioural control	19.1 ± 4.05	18.6 ± 5.03	0.720
Behavioural attention	65.5 ± 9.68	64.9 ± 11.1	0.628

* *p*-value was calculated using the Mann–Whitney U-test.

**Table 5:** Association between the behavioural attention scale and the sociodemographic characteristics of the participants (*n* = 385).

Factor	Behavioural Attention Score (100) Mean ± SD	H-Test	*p*-Value *
Age group			
16 years	65.1 ± 9.56	2.327	0.099
17 years	66.7 ± 9.90		
18 years	64.1 ± 10.3		
Educational region office			
South educational office	63.4 ± 9.62	28.813	<0.001 **
North educational office	67.2 ± 8.77		
West educational office	66.6 ± 9.71		
Central educational office	61.3 ± 10.0		
East educational office	68.4 ± 10.2		
Academic year level			
First year	66.1 ± 8.94	2.592	0.274
Second year	64.2 ± 9.80		
Third year	65.9 ± 11.0		

* *p*-value was calculated using the Kruskal–Wallis test. ** Significance at *p* < 0.05.

**Table 6:** Multiple mean comparisons between behavioural attention according to the educational region office.

(I) Educational Region Office	(J) Educational Region Office	Mean Difference (I–J)	Std. Error	Sig.	95% Confidence Interval	
					Lower Bound	Upper Bound
South educational office	North educational office	−3.80654	1.54127	0.140	−8.1583	0.5453
	West educational office	−3.18321	1.54617	0.402	−7.5489	1.1824
	Central educational office	2.12545	1.55631	1.000	−2.2688	6.5197
	East educational office	−5.02653 *	1.56692	0.015	−9.4508	−0.6023
North educational office	South educational office	3.80654	1.54127	0.140	−0.5453	8.1583
	West educational office	0.62333	1.54127	1.000	−3.7285	4.9751
	Central educational office	5.93199 *	1.55144	0.002	1.5515	10.3125
	East educational office	−1.21999	1.56209	1.000	−5.6306	3.1906
West educational office	South educational office	3.18321	1.54617	0.402	−1.1824	7.5489
	North educational office	−0.62333	1.54127	1.000	−4.9751	3.7285
	Central educational office	5.30866 *	1.55631	0.007	0.9144	9.7029
	East educational office	−1.84332	1.56692	1.000	−6.2676	2.5809
Central educational office	South educational office	−2.12545	1.55631	1.000	−6.5197	2.2688
	North educational office	−5.93199 *	1.55144	0.002	−10.3125	−1.5515
	West educational office	−5.30866 *	1.55631	0.007	−9.7029	−0.9144
	East educational office	−7.15198 *	1.57693	0.000	−11.6045	−2.6995
East educational office	South educational office	5.02653 *	1.56692	0.015	0.6023	9.4508
	North educational office	1.21999	1.56209	1.000	−3.1906	5.6306
	West educational office	1.84332	1.56692	1.000	−2.5809	6.2676
	Central educational office	7.15198 *	1.57693	0.000	2.6995	11.6045

Post-hoc test was performed using the Dunn–Bonferroni test. * The mean difference was significant at the 0.05 level.

## Data Availability

The data presented in this study are available on request from the corresponding author.
